# Buprenorphine treatment retention and comorbidities among patients with opioid use disorder in a primary care setting

**DOI:** 10.1111/ajad.13268

**Published:** 2022-04-06

**Authors:** Mary M. Sweeney, Laura Prichett, Michael I. Fingerhood, Denis Antoine, Annie Umbricht, Kelly E. Dunn, Megan E. Buresh

**Affiliations:** ^1^ Behavioral Pharmacology Research Unit, Department of Psychiatry and Behavioral Sciences Johns Hopkins University School of Medicine Baltimore Maryland USA; ^2^ Department of Pediatrics, Biostatistics, Epidemiology, and Data Management Core Johns Hopkins University School of Medicine Baltimore Maryland USA; ^3^ Department of Medicine, Division of Addiction Medicine Johns Hopkins University School of Medicine Baltimore Maryland USA; ^4^ Department of Mental Health Johns Hopkins Bloomberg School of Public Health Baltimore Maryland USA; ^5^ Department of Epidemiology Johns Hopkins Bloomberg School of Public Health Baltimore Maryland USA

## Abstract

**Background and Objectives:**

More information is needed about comorbidities among patients receiving buprenorphine maintenance treatment and their relationship with retention.

**Methods:**

Retrospective electronic health record data over a 5‐year period from primary care patients receiving buprenorphine for the treatment of opioid use disorder were examined (*N *= 899). The present analysis determined the prevalence of comorbidities and examined associations with treatment retention as defined by cumulative duration of buprenorphine prescription.

**Results:**

Tobacco use and comorbidities including hypertension were prevalent but did not predict retention according to survival analyses controlling for demographic characteristics. Retention was poorer among patients testing positive for cocaine (HR = 1.38, 95% CI: 1.09–1.74, *p =* .007) and patients with hepatitis C virus (HR = 1.17, 95% CI: 1.01–1.37, *p *= .04).

**Conclusion and Scientific Significance:**

This study provides new knowledge of previously unexamined associations between comorbidities (e.g., hypertension) and buprenorphine treatment retention. The robust association between cocaine use and poorer buprenorphine retention serves to resolve prior conflicting data in the literature.

## INTRODUCTION

Treatment of opioid use disorder (OUD) with buprenorphine, a medication prescribed directly from a clinician's office, has been associated with decreased opioid use and reduced mortality among persons with OUD.^1^ Providing buprenorphine in primary care provides an ideal opportunity to optimize the overall health of OUD patients with comprehensive care that simultaneously addresses OUD and other health comorbidities such as hypertension, human immunodeficiency virus (HIV), hepatitis C virus (HCV), and diabetes.^2^ Prior research favors integrating treatment with primary care over segregated addiction treatment models, with evidence to support greater satisfaction, lower associated medical costs, and higher retention among patients seen in comprehensive primary care.^2^, ^3^, ^4^ Even so, less than half of patients prescribed buprenorphine remain in treatment for a year or more,^4^, ^5^ which suggests the treatment could be further optimized and emphasizes the importance of identifying risk factors for attrition.

Although comprehensive care models including buprenorphine were designed to address patient comorbidities, few studies have described the health comorbidities of persons receiving buprenorphine in primary care and determined their relation to retention. Prior research has indicated that persons receiving buprenorphine in primary care settings who were female, younger, or had a psychiatric diagnosis had higher retention in treatment, whereas individuals who were African American, Hispanic, unemployed, or were HCV positive had decreased retention.^6^ Use of cocaine is robustly associated with poorer retention among patients receiving methadone pharmacotherapy for OUD, but data regarding cocaine use and buprenorphine treatment attrition are mixed.^7^ Some of the variability in prior findings associating cocaine use and buprenorphine attrition may be attributable to a smaller sample size or reliance on self‐report rather than biochemical verification of cocaine use.^7^ Several other important health indicators, including cardiovascular risk factors such as hypertension and obesity, have not been well‐characterized among patients receiving buprenorphine in primary care either in terms of prevalence relative to general primary care or relationship to retention.

Primary care models would benefit from additional information regarding the medical and psychiatric needs of persons receiving buprenorphine and increased knowledge of how comorbidities influence retention. These patient‐level data could help inform intervention development and optimization of interdisciplinary care. Thus, the present study evaluated electronic health record (EHR) data from patients receiving buprenorphine in primary care to identify common comorbidities and evaluate how patient comorbidities and physiological, demographic, or psychosocial characteristics relate to retention in treatment.

## METHODS

### Patient population

Patients attended a single‐site primary care clinic in Baltimore, Maryland, that integrates buprenorphine maintenance treatment for OUD with general primary care, including hypertension, diabetes, HIV, and HCV treatment.^3^ Patients undergo a comprehensive evaluation, including OUD assessment, instructions for home initiation of buprenorphine, general health and social history, and onsite urine drug screening. Patients attend the clinic through a variety of referral means including self‐referral and Emergency Department referral. Patients are typically insured by Medicaid/Medicare or employer health insurance plans.

### Data query and extraction

Deidentified data were extracted via bulk query from the EHR (Epic Systems) for all clinic patients who received ≥1 buprenorphine prescription between January 1, 2014, and 1 January 1, 2019. Baseline was defined as the first clinical contact during the query period where an order is placed for buprenorphine or the patient is reported as taking buprenorphine. Patient characteristics at baseline were extracted from the EHR, including demographics, blood pressure, body mass index (BMI), HIV and HCV status, psychiatric comorbidities, prescription medications, lifetime/current tobacco use (using specified EHR form fields), and baseline urine drug testing for cocaine. Other urine toxicology data (e.g., benzodiazepines) are routinely collected for clinical use but were not available in the EHR for the present analyses. Psychiatric comorbidities (e.g., anxiety) were queried using multiple codes in the electronic problem list (e.g., anxiety disorder, generalized anxiety disorder, panic disorder, etc.). Psychiatric medications included any on the Johns Hopkins EHR medication report and were not limited to those prescribed by clinic providers. The Johns Hopkins School of Medicine Institutional Review Board approved this study.

### Statistical analysis

Patient characteristics and buprenorphine treatment retention are presented descriptively. We calculated retention using first treatment episode during the query period, that is, first buprenorphine prescription start date and last prescription end date, encompassing all continuous prescriptions (i.e., until last end date, or last end date before a gap between prescriptions >30 days). Duration of buprenorphine treatment could be calculated using prescription start and end dates for 98% of the sample (*n* = 879). Multivariate Cox proportional hazard models controlling for age, sex, and race/ethnicity examined factors associated with buprenorphine retention “survival” for each variable. Persons whose active buprenorphine prescription retention outlasted the query period (*n* = 92) were included in the survival analyses and coded as censored using their number of days retained at the end date of the query period. Censored values were excluded from descriptive statistics for retention. There were no missing data for age or sex. Missing data included race/ethnicity (<1% declined to identify for both race and ethnicity; patients indicating other/unknown, 1%–2%, were included as non‐White), blood pressure (1%), buprenorphine prescription start/end date (2%), employment (4%), BMI (6%), and cocaine urine results (31%). Participants with missing data were excluded only for analyses using the missing variable. Comorbidities (e.g., depressive symptoms), tobacco use, and prescribed medications were dichotomized as confirmed present versus absent for all participants. Analyses were conducted using Stata 15.1.

## RESULTS

### Sample characteristics

#### Sample size and demographics

Patients (*N* = 899) were majority female (53%) and White (75%). The mean age was 43 years (SD = 12). Fifty‐three percent of the sample were not employed, 21% were employed full time, and 14% were disabled for employment. Overall, 12% of the patients tested positive for cocaine and 57% tested negative (31% missing cocaine urine results). Seventy‐eight percent of patients endorsed tobacco use.

#### Comorbidities

Table 1 shows baseline physiological data and prevalence of health comorbidities. Hypertension was the most common medical comorbidity (69%), followed by HCV (36%). Thirty‐three percent of patients were categorized as obese (BMI > 29.9) and only 3% as underweight (BMI < 18.5). Depressive (17%) and anxiety symptoms (12%) were the most common psychiatric comorbidities.

**Table 1:** Patient characteristics and buprenorphine treatment retention survival analyses

Patient sample characteristics (*N* = 899)			Retention survival analyses					
Variable	*M* (SD)	Count (%)	Hazard ratio	S.E.	*z*	*p*	L.L. 95% CI	U.L 95% CI
Age^a^	42.8 (11.6)		1.00	0.00	−0.21	.83	0.99	1.01
Female^b^		479 (53.3)	1.05	0.08	0.67	.50	0.91	1.21
Black^c^		184 (20.5)	0.95	0.09	−0.51	.61	0.79	1.14
Race/ethnicity other than White^d^		239 (26.6)	1.02	0.09	0.27	.79	0.87	1.21
Employed (full or part‐time)		226 (25.1)	0.94	0.08	−0.79	.43	0.79	1.10
Physiological data								
Hypertension		616 (68.5)	1.02	0.08	0.20	.84	0.87	1.19
BMI	28.2 (6.9)		1.00	0.01	0.83	.41	0.99	1.02
Obesity		295 (32.8)	1.11	0.09	1.42	.15	0.96	1.29
**Cocaine positive**		**109 (12.1)**	**1.38**	**0.16**	**2.68**	**.007**	**1.09**	**1.74**
Comorbidities and behaviors identified in clinical history								
Current or lifetime tobacco use		704 (78.3)	0.88	0.08	−1.51	.13	0.74	1.04
Diabetes		56 (6.2)	0.94	0.15	−0.40	.69	0.68	1.28
HIV		62 (6.9)	1.03	0.15	0.22	.83	0.78	1.38
**HCV**		**327 (36.4)**	**1.17**	**0.09**	**2.07**	**.04**	**1.01**	**1.37**
Depressive symptoms		155 (17.2)	0.96	0.10	−0.38	.70	0.79	1.17
Anxiety symptoms		104 (11.6)	1.14	0.14	1.09	.28	0.90	1.45
Bipolar disorder		48 (5.3)	1.07	0.17	0.43	.67	0.78	1.47
ADHD		33 (3.7)	0.93	0.17	−0.40	.69	0.64	1.34
Current prescribed medications								
Anti‐hypertensive		123 (13.7)	1.13	0.12	1.15	.25	0.92	1.40
Benzodiazepine		109 (12.1)	1.14	0.13	1.19	.24	0.92	1.42
Atypical anti‐psychotic		101 (11.2)	0.83	0.10	−1.60	.11	0.66	1.04
Anti‐depressant		51 (5.7)	0.87	0.14	−0.88	.38	0.64	1.18
Any psychiatric medication^e^		219 (24.4)	0.95	0.08	−0.59	.56	0.81	1.12

*Note*: Each line contains descriptive statistics (left), and treatment retention survival analysis (right) for that variable controlling for age, sex, and race/ethnicity unless otherwise specified. Bold indicates factor is significant at *p *< .05.

Abbreviations: ADHD, attention deficit hyperactivity disorder; BMI, body mass index; CI, confidence interval; HCV, hepatitis C virus; HIV, human immunodeficiency virus; L.L. and U.L., lower limit and upper limit for 95% confidence interval; S.E., standard error.

^a^ Controlling for sex and race/ethnicity.

^b^ Relative to male, controlling for age and race/ethnicity.

^c^ Relative to all other races, controlling for age and sex.

^d^ Any other race/ethnicity relative to White, controlling for age and sex.

^e^ Includes any of above listed medications or dopamine antagonist anti‐psychotics.

### Treatment retention

The mean duration of buprenorphine retention was 432.9 days (SD = 413.6; range: 2–1905 days) with substantial positive skew (skewness = 1.4). A substantial majority of patients (80%) were retained at least three months, 65% of patients were retained at least six months, and 43% were retained more than 1 year.

### Correlates of retention

Table 1 shows survival analyses controlling for covariates, where a hazard ratio (HR) < 1 means the factor is associated with longer retention. A cocaine‐positive urine test was strongly associated with poorer retention (mean = 337 days, SD = 318) relative to individuals who tested negative (mean = 457 days, SD = 415) for cocaine (HR = 1.38, 95% CI: 1.09–1.74, *p* = .007). Being HCV‐positive (HR = 1.17, 95% CI: 1.01–1.37, *p* = .04) was also associated with shorter buprenorphine treatment period.

## DISCUSSION

This study utilized EHR data to examine comorbidities among patients receiving buprenorphine in primary care and evaluate their relationship to treatment retention. Testing positive for cocaine was the strongest predictor of discontinuing buprenorphine. Significant medical comorbidities were prevalent, and among these HCV‐positive status was modestly but significantly associated with attrition. Further innovation is needed to improve retention and address health conditions among OUD patients.

In the present sample, 43% of patients were retained for 1 year, which is comparable to prior data, but emphasizes the importance of optimizing buprenorphine treatment retention through identification of patients at high risk for attrition.^4^, ^5^ The association of cocaine positive urinalysis results with treatment drop‐out provides additional weight to prior observations of cocaine use as a risk factor for treatment drop‐out, where some studies had observed null findings for cocaine, perhaps due to smaller sample sizes relative to the present analysis or use of self‐report data rather than toxicology to characterize cocaine use.^6^, ^7^ Because buprenorphine pharmacotherapy does not directly treat cocaine use disorder, and no pharmacotherapy currently exists to treat cocaine use, the present data suggest that targeted behavioral interventions among patients receiving buprenorphine who use cocaine are merited.

Examination of comorbidities found HCV‐positive status was modestly but significantly associated with treatment attrition, consistent with prior data.^6^ Although speculative, HCV may associate with attrition because it functions as an incomplete proxy for injection use/OUD severity. Comorbidities other than HCV were not significantly associated with retention, but they still impact patient quality of life. High rates of hypertension and tobacco use have also been observed among OUD patients receiving methadone.^8^ When compared with general primary care, it is noteworthy that some comorbidities, such as hypertension, were present more often in our sample (69%) than other primary care populations (27%),^9^ further supporting an integrated treatment strategy for patients with OUD.

This study has several strengths, including using patient‐level medical data and buprenorphine prescription duration information among more than 800 patients in a real‐world treatment setting and controlling for patient demographic characteristics. Nevertheless, these data have limitations. Differences in provider practices for documentation and use of free text rather than standardized EHR fields led to missing toxicology and social history data and may limit the sensitivity of associations between patient characteristics and treatment retention. Psychiatric comorbidities in the analyses were based on limited information in the EHR including symptoms or diagnoses and were not necessarily validated by structured clinical interviews. Further, the clinic is part of an academic medical center and is not representative of most office‐based buprenorphine clinics. Buprenorphine dose has been associated with retention in previous research but was not included in the present analysis. Our clinical experience is that most patients are prescribed at least 16 mg, thus it is likely that dose range was limited, and dose was not likely to be an informative covariate in this specific patient population. The study sample had a high proportion of women relative to other OUD treatment samples and was not racially and ethnically diverse. Evaluation of comorbidities among Black and Hispanic persons is especially warranted because prior research indicates buprenorphine treatment retention is poorer among these groups.^6^ Retention was defined using prescription data and was not biologically verified or monitored using film counts. We did not evaluate reasons for attrition including incarceration, death, or changes in treatment plan. Given that retention generally increases with repeated opioid pharmacotherapy treatment episodes, future research should evaluate the relationship between comorbidities and improvement in retention over repeated treatments.^10^ Finally, there are additional retention‐relevant factors and reasons for attrition that we did not evaluate, such as psychosocial support, home environment, injection drug use, buprenorphine dose, and additional urine toxicology results.

## CONCLUSIONS

Office‐based buprenorphine treatment presents an opportunity to deliver comprehensive interdisciplinary care. Cocaine‐positive toxicology results and HCV are readily identifiable markers for treatment attrition that providers should utilize to prioritize resources to prevent patient drop‐out. Buprenorphine providers should monitor and treat comorbid conditions so that successfully retained patients can experience additional therapeutic benefits, improved quality of life, and further reductions in morbidity and mortality.

## CONFLICTS OF INTEREST

The authors report no conflicts of interest. The authors alone are responsible for the content and writing of this paper.
